# Three Reconstructions of ‘Effectiveness’: Some Implications for State Continuity and Sea-level Rise

**DOI:** 10.1093/ojls/gqae003

**Published:** 2024-02-21

**Authors:** Alex Green

**Keywords:** statehood, state continuity, Small Island Developing States, sea-level rise, public international law

## Abstract

Small Island Developing States (SIDS) are uniquely threatened by rising sea levels. Not only does the retreat of their coastlines place them in danger of losing maritime territory; the concurrent possibility of their landmasses becoming either uninhabitable or completely submerged also threatens their very existence. According to one understanding of the law that governs the continuity and extinction of states, political communities that permanently lose ‘effectiveness’—typically understood as sufficient governmental control of a relatively determinate territory with a permanent population—must lose their statehood as well. In this article, I provide three reconstructions of effectiveness, each of which rests upon a different normative rationale. My contention is that, regardless of which reconstruction one adopts, the continuity of submerged SIDS is eminently supportable, notwithstanding the arguments frequently made in favour of their formal extinction.

## 1. Introduction

The existence of states under international law turns on a range of connected factors, including a strong presumption in favour of continuity once legal statehood is established.^1^ When it comes to state creation, relevant factors include the presence of foreign recognition,^2^ the delimiting influence of treaties making territorial concessions,^3^ the express or implied consent of any ‘parent’ states,^4^ demonstrable commitments to democratic principles and human rights norms,^5^ and the provision of suitably constituted independence referendums at the point of emergence.^6^ It is arguable that some of these factors, particularly that of foreign recognition, also govern the existence of states beyond the point of their creation.

Whatever the case, one concept almost always discussed whenever statehood is in question is that of ‘effectiveness’. Traditionally conceived, effectiveness concerns whether a given physical space and human population are subject to factual control by the governance institutions that partly constitute the state in question.^7^ It is often considered necessary for the creation of states,^8^ in addition to being an independent basis upon which territorial title can be grounded.^9^ This article partly concerns the nature of effectiveness in general. However, my primary focus is upon the role that it plays within the law of state continuity, which governs the conditions under which states persist through time. The antithesis of continuity is extinction, which usually occurs due to some disruptive event, such as destruction by a foreign power or voluntary dissolution.^10^ States are continuous to the extent that their existence under international law is not disrupted by events of this kind. State continuity is sometimes linked with the neighbouring question of state identity, which concerns whether (and why) a state at time T1 is the same entity as the one identified with it at time T2.^11^ These topics can nonetheless be treated separately, which is what I propose to do here.^12^

My analysis of effectiveness is partly theoretical, turning upon three distinct accounts of that concept and what each has to say about state continuity. However, my motivation is practical, stemming from the existential threats currently faced by Small Island Developing States (SIDS) in light of human-caused sea-level rise.^13^ I aim to show that even though the three ‘reconstructions’ of effectiveness I advance have different normative foundations, each one supports the existential resilience of SIDS notwithstanding the danger of sea-level rise. That danger might be crudely described as ‘loss of effectiveness’. Under austere accounts of the effectiveness principle, no entity without inhabitable land and a permanent population living upon that land can maintain statehood, particularly not if the loss of these factual prerequisites is permanent.^14^ I elaborate upon this ‘austere view’ below, arguing that each reconstruction of effectiveness I examine requires it to be rejected.

All three accounts of effectiveness I advance are derived via the ‘rational reconstruction’ of international law.^15^ This hermeneutic method, sometimes called ‘creative’ or ‘constructive’ interpretation,^16^ seeks to induce from the social facts of international legal practice the set(s) of general evaluative commitments underpinning that practice.^17^ ‘Practice’, in the relevant sense, encompasses not only the state practice and *opinio juris* necessary for the formation of customary international law,^18^ but also the text and context of relevant treaties, the judgments of international courts and tribunals, and other international legal instruments with probative value on *de lege lata*.^19^ What distinguishes rational reconstruction from purely doctrinal legal interpretation is that it also relies upon ‘critically normative’ or ‘moral’ considerations to explicate the justificatory basis of the legal positions being examined.^20^ It takes social practices like international law seriously as sources of genuine practical reasons,^21^ and elucidates those reasons to yield prescriptive implications specific to these practices.^22^ Rational reconstruction, to that extent, exemplifies the ‘Grotian tradition’ of international law, as articulated by those such as Lauterpacht, and can be understood largely in those terms.^23^ The value of examining effectiveness in this way lies not only in the radical potential of rational reconstruction to generate progressive legal arguments,^24^ but also in its capacity to draw out the most foundational commitments of the international legal order. By asking why effectiveness matters in normative terms, we get a clearer picture of how it should be understood and applied in response to unprecedented legal challenges such as those of sea-level rise and the global climate crisis.

To provide context, section 2 introduces the most commonly accepted elements of effectiveness and connects them to other aspects of the law governing state continuity. After this, three discrete reconstructions of effectiveness are advanced, each corresponding to a different conception of why effectiveness matters normatively. The first emphasises the value of stability within international relations (‘effectiveness as stability’, section 3). The second focuses upon the function of governments as fiduciaries for their people, emphasising the connection between effectiveness and the protection of human rights (‘the fiduciary model’, section 4).^25^ The third stresses the importance of states as the primary communities within which intrinsically valuable political action occurs (‘statehood as political community’, section 5).^26^ Sections 3–5 are each divided into two halves: a normative reconstruction of effectiveness, followed by an application of that analysis to state continuity and sea-level rise. I conclude by reviewing the contribution of all three reconstructions. To the extent that each has featured within legal scholarship before, all three are typically presented as incompatible competing reconstructions.^27^ I engage with them here on a different basis: as distinct but compatible conceptions of effectiveness, each of which reinforces the existential resilience of SIDS under contemporary international law.

## 2. Effectiveness and State Continuity: Preliminaries from Practice

Traditional understandings of effectiveness, premised on the basic facticity of statehood, can be found across international legal practice. The arbitral award in *Deutsche Continental Gas-Gesellschaft v Polish State* provides as follows: ‘In order to say that a State exists … it is enough that [its] territory has a sufficient consistency, even though its boundaries have not yet been accurately delimited, and that the State actually exercises independent public authority over that territory.’^28^This notion of a core territory, guaranteed by the actual exercise of independent public authority, is widespread. It was mirrored within opinion No 1 of the Badinter Commission, which held: ‘the State is commonly defined as a community which consists of a territory and a population subject to an organized political authority’.^29^ Similarly, it is given extended treatment by the Commission of Jurists, appointed by the Council of the League of Nations in 1920 to report on the legal aspects of the Åland Islands dispute, which found that:

In the midst of revolution and anarchy, certain elements essential to the existence of a State … [are] lacking … Political and social life [is] disorganized; the authorities [are] not strong enough to assert themselves; civil war [is] rife … [state creation in circumstances of secession cannot be established] until a stable political organization had been created, and until the public authorities … become strong enough to assert themselves throughout the territories of the [new] State without the assistance of foreign troops … [only then is it] possible to re-establish order and normal political and social life, little by little.^30^

Perhaps most famously, ‘statehood as effectiveness’ is expressed within Article 1 of the 1933 Montevideo Convention on the Rights and Duties of States, which reads as follows: ‘The state as a person of international law should possess the following qualifications: (a) a permanent population; (b) a defined territory; (c) government; and (d) capacity to enter into relations with other states.’

Effectiveness is sometimes elided with Article 1(c), in the form of a statehood criterion of ‘effective government’.^31^ This is perhaps because, on traditional accounts, that criterion requires merely ‘some degree of maintenance of law and order and the establishment of basic institutions’.^32^ Nonetheless, effectiveness in its broadest sense refers to the first three Montevideo criteria: the notion of a government that maintains law and order over a more-or-less defined territory and a permanent population.^33^ (The fourth Montevideo criterion, the capacity to enter into relations with other states, is often treated as a consequence of statehood rather than as a prerequisite for that status.^34^) To these are commonly appended the further criterion of relative political independence, which is best understood as the absence of inter-governmental domination.^35^ Colonies and puppet states lack this quality, whereas uncontroversially established states, like the French Republic or Tuvalu, are demonstrably not subject to the formal or *de facto* domination of any foreign government.

‘Factual effectiveness’, as I shall call it, characterises Article 1 of the Resolution Concerning the Recognition of New States and New Governments, adopted by the Institut de Droit International in April 1936, which emphasises ‘the existence on a definite territory of a human society politically organized, independent of any other existing State, and capable of observing the obligations of international law’.^36^ It is also reflected within Article 3(ii) of the Draft Articles on the Law of Treaties, presented in 1956 to the International Law Commission (ILC), which defined statehood as ‘consisting of a people inhabiting a defined territory, under an organized system of government, and having the capacity to enter into international relations binding the entity as such, either directly or through some other State’.^37^ I take factual effectiveness, and the various elements of international practice that establish its legal content, as a non-exhaustive point of departure: none of the reconstructions considered below would be plausible without ‘fitting’ it to a considerable degree.^38^ The issue, as I demonstrate below, is what might justify its emphasis upon factual control and what this might imply for the capaciousness of effectiveness in general, once those reasons are made clear.

Like all principles of international law, effectiveness must be understood within its legal context. In terms of state continuity, I follow Crawford in holding effectiveness to condition the persistence of states across time, in that any political community that loses effectiveness completely will also face the loss of statehood.^39^ However, this position is nuanced by international law’s strong presumption of state continuity, which entails that even prolonged periods of ineffectiveness will not result in statehood lapsing.^40^ Moreover, each reconstruction I examine below emphasises the embeddedness of effectiveness within an even larger context. Effectiveness as stability, for example, focuses on the interaction between factual effectiveness and principles such as the immutability and intangibility of boundaries, whereas the fiduciary model emphasises the connection between effective government and the protection of human rights, and statehood as political community focuses on the state as a site for collective self-determination. On this basis, the real question *vis-à-vis* effectiveness is not *whether* it matters for state continuity but *what* it requires, given the particular context(s) of climate change and sea-level rise.

Before turning to these reconstructions, it pays to establish the contours of the presumption of continuing statehood within international legal practice, given its limiting effect upon factual effectiveness within the law of state continuity. The presumption is used by some to explain why even very lengthy lapses in government, and considerable losses of both territory and population, have been treated as not derogating from state continuity.^41^ There is considerable sense to this. The continuity of states such as the Democratic Republic of the Congo (DRC) or the Syrian Arab Republic can *only* be explained by the operation of that presumption, given their lengthy lapses of governmental control.^42^ Furthermore, the alleged creation of Slovakia under German occupation in 1939 did not extinguish the Kingdom of Yugoslavia, which maintained a government in exile until the creation of the Socialist Federal Republic of Yugoslavia (SFRY) in 1945.^43^ Even in cases of extreme territorial change, such as Poland from 1939 to 1945, continuity was never truly doubted.^44^

Nonetheless, the presumption of state continuity should not be overestimated. States can and do become extinct where their losses of population, territory, government or independence have been total or permanent.^45^ Moreover, in every recorded case where statehood has endured notwithstanding considerable disruption, the cause(s) of disruption have been wholly geopolitical, such as unlawful annexation or belligerent occupation.^46^ By contrast, sea-level rise is primarily geophysical, and therefore presents an unprecedented risk with potentially permanent implications.^47^ In terms of contemporary practice, there are several indications that the presumption of continuing statehood extends to such cases. At the 2018 International Law Association Conference, ‘it was generally agreed that, as guidance and as a starting point, there should be a presumption of continuing statehood in cases where land territory was lost’.^48^ Similarly, in the Sixth Committee of the United Nations (UN) General Assembly in October 2021, several states made submissions in favour of a very strong presumption in circumstances of sea-level rise.^49^ The Independent State of Samoa, speaking for all Pacific SIDS, stated that ‘Under international law, there is a presumption that a State, once established, will continue to be a State, particularly if it has a defined territory and population, among other factors’.^50^ Most recently, the Pacific Islands Forum released a Declaration on 9 November 2023 stating:

international law supports a presumption of continuity of statehood and does not contemplate its demise in the context of climate change-related sea-level rise … [such that] the statehood and sovereignty of Members of the Pacific Islands Forum will continue, and the rights and duties inherent thereto will be maintained, notwithstanding the impact of climate change-related sea-level rise.^51^

Set against this practice is the ‘austere view’ of state continuity.^52^ This holds that no state can exist without territory, and territory necessarily implies at least some inhabitable land. Scholars such as Crawford can be read as supporting this view, holding ‘that statehood implies exclusive control over *some* territory, small or large’ [emphasis in original].^53^ Similar remarks were made by Oppenheim, who held that ‘a State without a territory is not possible’,^54^ and Jessup, who averred ‘that one cannot contemplate a State as a kind of disembodied spirit’.^55^ Perhaps the clearest endorsement comes from the Badinter Commission, which held that ‘the existence or disappearance of the State is a question of fact’,^56^ which it understood, as Nicholson observes, entirely in terms of factual effectiveness.^57^ As Shaw notes, some version of the austere view is at least as old as Plato, although it was not always as popular as it seems to be now.^58^ The austere view also appears within several recent state submissions to the ILC,^59^ and within the UK it has also been adopted by the House of Lords International Relations and Defence Committee.^60^ In what follows, I argue that the best available reconstructions of effectiveness suggest that the austere view is mistaken and that a more liberal view of state continuity should be preferred.

## 3. Effectiveness as Stability

My first reconstruction holds effectiveness to be motivated by a concern for peace and stability within international relations. According to ‘effectiveness as stability’, both factual effectiveness and the presumption of continuing statehood should be read as constitutive parts of a broader ‘stability meta-principle’, which grounds and renders normatively coherent several aspects of international law. This view can be located, for example, within Ratner’s claim that the ‘thin justice of international law’ is primarily concerned with the promotion of peace,^61^ and in Capps’s emphasis upon the stability value of factual effectiveness when it comes to state creation.^62^ It can be found also in the arguments of those such as Oppenheim and Weil, who suggest international law in general is justified by its ability to forestall, and to resolve swiftly, international disputes.^63^ In this section, I begin by situating both effectiveness and the presumption of state continuity within the stability meta-principle and then show how reading effectiveness in light of that meta-principle should lead us to conclude that states can endure notwithstanding the complete loss of either inhabitable land or territory as such.

### A. Factual Effectiveness and the Stability Meta-principle

The hallmark of effectiveness as stability is instrumentalism.^64^ In favour of factual effectiveness governing state creation, Ratner contends that

it makes sense to allow entities to take advantage of rules that promote peace amongst states as soon as they become states, and not wait until they are either recognized by other states, or meet some additional criteria for participation (or both).^65^

Moreover, in circumstances where ‘secessions seem irreversible … a norm that requires all states to treat such entities as states immediately upon achievement of the Montevideo criteria advances peace’.^66^ According to Ratner, this holds because the legal entitlements usually contingent upon statehood incentivise states to act within the confines of international law.^67^ This echoes Koskenniemi’s claim that states are more likely to comply with law that they have a hand in creating, since the capacity to create international law typically follows from statehood itself.^68^

Such claims about the stabilising potential of international law have some empirical basis,^69^ even if they are not uncontroversial.^70^ Ratner himself points to the inequality exclusion endemic with the pre-1945 order, arguing that ‘The apparent durability of that system … should not belie its profound instability and the extent to which it prevented the possibility of world peace’.^71^ Given that ‘world peace’ currently seems as far off as ever, readers may feel sceptical about this line of argument. For present purposes, however, I take it as read that international law has some kind of stabilising influence and that it is both plausible and useful to interpret and apply effectiveness to further that end.^72^

To this gloss upon the relationship between effectiveness and stability we might be tempted to append Capps’s claim that the former should be read in purely factual terms to prevent states from ‘blipping in and out of existence’, as they might were their legal status contingent, for example, upon compliance with international human rights norms.^73^ However, factual effectiveness itself quite often fluctuates, particularly in response to episodes of armed conflict. In addition to the above-noted examples of the DRC and Syria, the 2014 invasion of Crimea and the 2022 invasion of Ukraine demonstrate viscerally how governmental control can fluctuate in response to illegal intervention.^74^ To these we might add the ongoing civil wars within the Central African Republic and the instability within the Federal Republic of Somalia as just two more examples. All five states nonetheless enjoy widespread recognition, such that their statehood is not really in doubt. The often-fragile nature of factual effectiveness indicates a tension within stability-focused conceptions: making statehood contingent upon factual control *may* increase stability, but it need not. As Ratner notes,

treating an entity emerging from secession as a state as soon as its government achieves effective control over territory and population may prevent a restoration of the status quo ante that would actually restore peace. It would also encourage future secessions elsewhere … —‘once you get control, you’re a state’.^75^

Fortunately for stability-focused conceptions, effectiveness does not operate in a vacuum. International law forbids state creation via the unlawful use of force alone,^76^ and the territorial integrity of established states creates a significant normative hurdle that must be overcome, in most cases, before new states can arise.^77^ Moreover, and however its merits should be judged in the round,^78^ the principle of *uti possidetis iuris* as applied to territorial boundaries secured some measure of stability during decolonisation by ‘prevent[ing] irredentist tendencies by neighbors from turning into territorial claims and the possible use of force’.^79^ Moreover, although its effects within more recent practice have been similarly mixed, ‘the certainty of the location of a new border … prevent[s] actors during secessions and dissolutions from trying to change it forcibly’.^80^ Finally, in terms of state continuity, the presumption of continuing statehood clearly possesses a stabilising function:

there are no obvious gains to be made in terms of international stability by an automatic revocation of statehood in response to domestic anarchy. This suggests that continuing statehood should be presumed unless there are strong (stability-related) reasons for holding otherwise. This argument seems adequate to explain the reluctance of international practice to accept the loss of statehood once that status has accrued, at least in very many cases …^81^

Every principle just listed—the prohibition on the threat or use of force,^82^ territorial integrity, *uti possidetis* and the presumption of continuing statehood—operates to contour and soften factual effectiveness, restricting its application. This has two implications from a stability-focused perspective. First, it allows us to identify a ‘stability meta-principle’ within the law of statehood, which operates at a more abstract level than the concrete legal norms just discussed, rendering them a more-or-less coherent set.^83^ That meta-principle also arguably extends to (or at least coheres with) several other areas of international law, such as that regulating the use and threat of force. For instance, the principle of immutability and intangibility of boundaries is described explicitly in these terms by the International Court of Justice (ICJ):

In general, when two countries establish a frontier between them, one of the primary objects is to achieve stability and finality. This is impossible if the line so established can, at any moment, and on the basis of a continuously available process, be called in question … Such a frontier, so far from being stable, would be completely precarious.^84^

To take another example, the United Nations Convention on the Law of the Sea (UNCLOS) is shot through with stability-related concerns.^85^ Its preamble states its underlying rationale to include ‘the maintenance of peace, justice and progress for all peoples of the world’, while its regime of compulsory dispute settlement operating under Part XV, and in particular the obligation to settle disputes by peaceful means under Article 279, showcase the importance it places upon stability.^86^ In light of these, and other potential examples, the heuristic case for a stability meta-principle is solid.

The second, and more radical, implication is that not only should the *application* of effectiveness be read in light of stability, but its *normative content* might also be understood with reference to that overarching aim. Factual effectiveness alone struggles to explain the continuity of states such as Somalia and the creation of states such as Bosnia and Herzegovina, which received widespread recognition that some considered ‘premature’.^87^ However, this practice starts to make more sense when reconstructed through the lens of stability. Recognition itself can have a stabilising role,^88^ which may go some way to justifying accounts of international law that regard it to be wholly or partly constitutive of statehood.^89^ Moreover, in cases like Bosnia and Herzegovina, the dissolution of the parent state,^90^ combined with the stability value of *uti possidetis*, would seem to make the demonstration of factual effectiveness less instrumentally important *vis-à-vis* stability. Absent the stability meta-principle, instances of ‘premature’ recognition and near-fictious instances of state continuity might seem legally *ad hoc*. But assuming the existence of that principle, our understanding of effectiveness might require more nuanced specification, whereby ‘factual control’ denotes merely some relevant circumstances picked out by that concept and not a full and accurate rendition of how it operates *as a legal principle*. Specifically, we might argue ‘the effectiveness principle’ to hold that *factual* effectiveness is sufficient but not necessary for both the creation and continuity of states,^91^ and that this holds only because, and to the extent that, international stability is promoted. This move fixes the meaning of effectiveness holistically in relation to that of other legal principles, such as *uti possidetis* or Article 2(4) of the UN Charter, rendering it a component part of larger legal whole.^92^ In explanatory terms, it fits not only cases such as Bosnia and Herzegovina, the DRC and Somalia, but also the collective non-recognition of, for example, the Donetsk or Luhansk People’s Republics, or Daesh,^93^ where the legitimation of factually effective entities would undermine peace.

### B. Stability, Inhabitable Land and Territory

Whether one adopts this more radical stability-based reconstruction or not, accepting the stability meta-principle has significant implications for state continuity in circumstances of sea-level rise. To recapitulate briefly, the dominant ‘austere view’ holds that: (i) effectiveness always requires relatively determinate territory; and (ii) this necessarily entails at least some inhabitable land. In this subsection, I argue that stability does not support these propositions. Instead, it favours state continuity even when all inhabitable land has been lost to rising sea levels. This holds for two reasons: first, very few stability-related considerations insist upon the connection between territory and inhabitable land; and second, it is by no means clear that a stability-focused account necessarily require states to exist as territorial entities.

Taking the first, maritime territory has long been recognised under international law, even though contemporary practice characteristically ‘anchors’ it to inhabitable land.^94^ But this practice, often characterised in terms of ‘the land dominates the sea’, belongs to the law of the sea, not to that of statehood.^95^ This matters principally because the law of statehood must be taken to possess ‘local priority’ on questions of continuity and extinction.^96^ Although stability is enhanced by relatively determinate and enduring territorial units, it does not follow that such territory must be land-based. Territory *could* be delineated without reference to land, as recently contemplated by the ILC, which observed that ‘sovereignty refer[s] to the whole territory under the State’s control and not solely to the land territory. Thus, a territory that became fully submerged because of sea-level rise should not be considered a non-existent territory.’^97^

In practical terms, this might be facilitated by ‘fixing’ maritime baselines: a process states can undertake by submitting copies of official charts to the UN Secretary-General under UNCLOS, Articles 16 and 47, and then refraining either from depositing further charts or from altering the position of their baselines in any subsequent charts.^98^ Once fixed, baselines might delimit maritime territory ‘seaward’ in the ordinary manner, resulting in a territorial sea and exclusive economic zone.^99^ Conversely, any sea ‘landward’ of the baseline would count as internal waters,^100^ or (where applicable) ‘submerged land’ territory.^101^ An alternative would be for baselines to be deemed ‘ambulatory’ in relation to the relevant state’s changing low-water mark,^102^ but for the outer limits of maritime zones to be fixed.^103^ This would result in gradually expanding territorial seas as baselines moved landward, over which other states would have rights of innocent passage.^104^

That UNCLOS in general holds baselines to be fixed or that it would permit the fixing of outer limits are both legally controversial.^105^ Nonetheless, several SIDS are pushing for global adoption of fixed baselines in response to sea-level rise,^106^ with the recent support of the United States of America.^107^ Whether or not they are ultimately successful in securing international consensus, the mere availability of this option demonstrates the practical cogency of territories delimited without reference to inhabitable land. As such, the view that such land *must* feature within any coherent conception of territory requires an additional premise beyond a commitment to readily identifiable boundaries. From the perspective of stability, it is not clear what that might be.

One such premise might be the empirical claim that purely maritime territory cannot be governed as securely as land. There is a danger, or so the argument might go, that asking SIDS to maintain control over large stretches of ocean without any logistical support from an adjacent landmass may result in areas of nautical anarchy.^108^ This is a serious point, but ultimately unconvincing. As noted above, many states have benefited from the presumption of continuing statehood notwithstanding considerable and protracted anarchy, often amounting to civil war, within their borders. This alone indicates that international law is committed to enduring even significant unrest without disturbing the *status quo*. Moreover, most submerged SIDS maintaining maritime territory would be appurtenant to the high seas. Conversely, states such as the DRC or Somalia are surrounded by other political communities, making endemic violence within their territories far more dangerous to international peace, relatively speaking. If global stability does not call statehood into question in these established cases, it should not in the case of submerged SIDS either. Connectedly, it can hardly be claimed that the high seas themselves are hotbeds for global unrest, even though no state possesses enforcement jurisdiction over them.^109^

A similar claim might be levelled against the idea of states without territory. Sharon argues that permitting the continuity of wholly non-territorial SIDS would undermine ‘the fundamental concept of the state in international law’.^110^ Moreover, he argues that were their diasporic populations ever to reside within a particular area *en masse*, perhaps through the leasing, conditional gifting or sale of land within the territory of another state, the continued existence of such SIDS would imperil the sovereignty of the host.^111^ This objection seems wrongheaded. Sharon plausibly asserts that if ‘the state continues to exist indefinitely, regardless of a link to a specific territory, then it exists for the people *wherever they are*’ [emphasis in original].^112^ However, he then seeks to establish the potential threat by assuming that *all* states necessarily make territorial title claims. But this begs the question because that assumption is precisely what non-territorial conceptions of statehood deny.^113^

In any event, there is no real prospect of diasporic populations threatening the territory of an established state by a unilateral claim of right, even with their own statehood intact. First, it would not be in their interests to do so, as any hint of that possibility would, as Sharon himself observes, massively disincentivise host states.^114^ Second, non-territorial SIDS would almost certainly be precluded from asserting sovereign claims in relation to territory over which they had explicitly accepted mere proprietary title,^115^ whether that preclusion operates by dint of treaty or through implied recognition and estoppel.^116^ Although the factual control that ‘relocated’ SIDS *may* eventually assert provides one basis for asserting territorial title,^117^ such control is typically trumped by contrary claims of right.^118^ Third, there have been no cases (outwith decolonisation) since 1945 where the UN has accepted the membership of a seceding entity against the wishes of its parent state.^119^ Secession and title claims by established states are distinct;^120^ however, this practice indicates that widespread recognition of ‘invasive’ title claims would be extremely unlikely.

An objection of another kind is that either option contemplated above in relation to autonomous maritime territory would cause significant disparities between the maritime zones of submerging states and their physical coastlines, resulting in internal waters or territorial seas far larger than those contemplated by UNCLOS, and in any event not dependent upon appurtenant coastlines, as envisaged by that treaty.^121^ This presents legal and logistical challenges, but no obvious issues of stability. We must not conflate the importance of peace and friendly relations with another sense in which ‘stability’ is sometimes used by international lawyers: that of the constancy of legal norms across time.^122^ Although stability of this sort may be conducive to international peace, it does not follow that every disruption to the legal *status quo* threatens global law and order. True, the amendment or reinterpretation of UNCLOS may result in international disputes; however, the current ambiguity of UNCLOS on whether baselines are fixed or ambulatory is scarcely less conducive to disagreement.^123^

Set against these arguments is one very weighty reason to suppose that stability favours either: (i) interpreting effectiveness so as to admit states without inhabitable land and/or territory; or (ii) adopting an effectively irrebuttable presumption of state continuity notwithstanding any lack of factual effectiveness. This is that any loss of statehood on the part of SIDS would render their populations legally stateless. Although deleterious in its own right (see below), statelessness also has major implications for peace and security. This has both internal and external dimensions. Taking the first, as per the High Commissioner on National Minorities of the Organization for Security and Co-operation in Europe:

Citizenship is without a doubt a most delicate question both legally and politically, in general terms and with regard to its denial or deprivation. The refusal to grant citizenship to a large number of titular residents may severely affect the balanced integration of all groups in society. Thus, it may represent a security threat.^124^

In brief, statelessness characteristically causes economic, cultural and social exclusion,^125^ which, when experienced in sufficient volume and intensity, can produce social unrest.^126^ Since nationality assumes statehood,^127^ prudence recommends the legal continuity of uninhabitable SIDS at least until their erstwhile populations gain dual or replacement nationality (assuming, of course, that sufficient numbers desire this).^128^

Turning to the external dimension, leaving aside that state extinction as such is rightly conceived by SIDS to be an ‘existential security risk’,^129^ the sheer scale of potential statelessness is key internationally. The current global population of SIDS is around 65 million (more than six times the total number of individuals currently experiencing statelessness).^130^ Naturally, not all populations affected by sea-level rise will become diasporic at once.^131^ However, even a steady trickle from such a vast number would risk causing serious co-ordination failures. To quote Stewart:

Even if massive population movement doesn’t lead to conflict, it can destabilize the existing balance of political power. For example, there are already connections being drawn between migration in the context of climate change and the rise of far-right nationalistic political parties.^132^

Even internal conflicts, when sufficiently intense and widespread, can become matters of international concern. Maintaining the statehood of sunken SIDS would not remove these risks altogether: the physical reality of creeping uninhabitability will occasion whatever migration it may. Nonetheless, state continuity would ensure ongoing governmental representation for diasporic populations, facilitating greater co-ordination between submerged and host communities.^133^ Moreover, as argued below, the endurance in law of both statehood and government would leave displaced individuals far better off than formal statelessness in terms of international legal protections, which would almost certainly reduce the feelings of political and cultural exclusion that often ferment social unrest. Taken together, these points ground a strong stability-focused case for effectiveness to be understood and applied in a manner consistent with the existential continuity of SIDS.

## 4. The Fiduciary Model

My second reconstruction emphasises the connection between effective government and human rights. It is grounded upon the view that states operate as fiduciaries.^134^ Their purpose, according to this view, is to secure the rights of every individual within their legislative, adjudicative and enforcement jurisdictions.^135^ Fiduciary conceptions are not without difficulties. First, if states were truly constituted to ensure that all individuals receive an equal degree of care and protection, we would expect to see far greater parity between them in terms of population size, territorial extent and the distribution of natural resources than is currently the case. Second, fiduciary conceptions tend to conflate states with governments.^136^ International practice consistently differentiates between the two,^137^ with fundamental constitutional changes and even revolutions not necessarily disrupting either continuity or identity.^138^ It is almost trite to hold governments to be the fiduciaries of their states, given their representative function under international law.^139^ By going one stage further, fiduciary conceptions of the state obscure the constitutive relationship between government *and* population on the one hand and statehood on the other, acknowledgement of which is arguably necessary for any defensible conception of effectiveness. This distancing of statehood from its constituent elements can be seen, for instance, in Criddle and Fox-Decent’s claim that ‘people and territory are not properly understood as “qualifications” for statehood, as the Montevideo Convention suggests, but rather as structural features that are common to all state-subject relationships’,^140^ and in Waldron’s deliberate shift, when analysing subjecthood and the international rule of law, from states, to state institutions, to governments.^141^ Nonetheless, fiduciary conceptions remain popular amongst academic lawyers with broadly liberal or republican leanings,^142^ as well as with political philosophers.^143^ As a result, I propose to take them seriously: I begin by establishing the connection between effectiveness and human rights protection, before asking whether wholly uninhabitable SIDS might continue to fulfil this function.

### A. Statehood and Human Rights

Following the Cold War, the European Community Guidelines on the Recognition of New States in Eastern Europe and the Soviet Union directed Member States to offer recognition to emerging communities only if they evidenced sufficient ‘respect for the provisions … in the Charter of Paris, especially with regard to … human rights’.^144^ The Charter of Paris itself provides three lists of rights, to be maintained without discrimination. The first includes the freedoms of expression, association, peaceful assembly and movement. The second lists the freedoms from arbitrary arrest and detention, and torture or other cruel, inhuman or degrading treatment or punishment. The final list references the right to know and act upon one’s rights, as well as the right to free and fair elections, fair and public trials, the ownership of property and the right to engage in ‘individual enterprise’. This list ends with a general commitment to the protection economic, social and cultural rights.^145^ On this basis, some authors contend that indications of future compliance with human rights norms are either a distinct criterion for state creation or form elements of effectiveness itself.^146^ In a similar vein, others have claimed that the collective non-recognition of the Turkish Republic of Northern Cyprus, Southern Rhodesia, and the Bantustans are best explained in terms of the serious human rights violations effected by the emergence of those entities.^147^

Support for prospective human rights compliance as a criterion for state creation is not universal.^148^ Moreover, there can be no serious suggestion that it also conditions the continuity of states: if this were so, political communities would be forever ‘blipping in and out of existence’ as their overall compliance fluctuates.^149^ Nevertheless, there is an importance sense in which ‘effective statehood’ is contingent upon human rights protection. To quote Criddle and Fox-Decent,

international law entrusts states with the responsibility to establish and maintain legislative, executive, and judicial institutions to provide basic public security, social services, and the rule of law for their people. States that decline to serve these functions undermine their own international legal authority to exercise public powers.^150^

To put this another way, states which violate human rights norms not only fail to act in a legally and morally acceptable manner: they also fail to act *as states characteristically should*, when statehood is understood as an ideal type from the fiduciary point of view.^151^ In response, rather than remove statehood completely, the fiduciary model recommends corrective limitations on sovereignty through temporary suspensions of some rights ordinarily associated with statehood, such as participation in international organisations or territorial inviolability.^152^ In this manner, according to Capps:

Statehood is determined juridically on criteria of [purely factual] effectiveness, but such states act illegally when failing to comply with international legal norms which reflect the moral concept of statehood. Thus, the moral concept of the state is employed to devise normative standards which are applied by international legal institutions. States … are held to account against such norms.^153^

Although Capps reserves the language of effectiveness for factual control, his ‘moral conception of statehood’ discloses a uniquely fiduciary answer to the question of what ‘effective statehood’ might mean. States failing to maintain ‘a regime of secure and equal freedom for their people’^154^ are ineffective insofar as they fail to instantiate statehood understood as an ideal type. This matters, as I now argue, because the fiduciary model necessarily implies the inverse as well. Where states continue to function as fiduciaries for their populations, they merit full sovereignty: that is, treatment as an entity with the rights characteristic of statehood under contemporary international law.^155^ Crucially, this holds notwithstanding the fact that such states may lack either inhabitable land or territory as such.

### B. Submerged Fiduciaries and the Protection of Persons

Hitherto, most proponents of the fiduciary model have assumed otherwise. For instance, Criddle and Fox-Decent contend that ‘International law distributes sovereignty primarily by dividing dominion over the earth’s surface geographically among states. States are charged with establishing municipal legal order for those who reside or travel within their territory’,^156^ while Sellers defines states as ‘political units, controlling a determinate territory’, and peoples as ‘the inhabitants of the different states’.^157^ The reason for this is clear: inhabitable territory provides an important resource for the protection of persons.^158^ Various legal principles emphasising the protective role of states, such as non-refoulement or the presumptively territorial nature of jurisdiction within international human rights law,^159^ assume this connection between physical refuge and legal protection. Most obviously, without some manner of inhabitable space within their *de facto* control, states cannot guarantee their populations rights of residence or return.^160^

Nonetheless, accepting that it is *easier* for states to act as fiduciaries where they possess title to at least some inhabitable land does entail that without such title states would completely lack that capacity. As Talmon attests, ‘one of the most noble tasks of States’ is the protection of their nationals abroad:^161^ a task which is necessarily extraterritorial. Short of military action, such protection characteristically requires ‘resorting to diplomatic action or international judicial proceedings’.^162^ In the first instance, assuming that domestic remedies have been appropriately exhausted,^163^ this typically occurs via diplomatic or consular missions within the host state.^164^ The capacity for such intervention exists for two intermediary legal reasons. First, at least as traditionally conceived, an injury to the national of a state abroad is an injury to that state itself.^165^ Second, notwithstanding the territorial basis of the host state’s jurisdiction, the injured state retains personal jurisdiction over its nationals abroad.^166^ Since diplomatic or international legal action can resolve many violations of such rights without requiring individual complainants to seek refuge within their home state, the presence or absence of inhabitable territory makes no practical contribution to the action of the intervening community.

Both the existence of diplomatic or consular missions and the voluntary nature of international adjudication nonetheless require ongoing diplomatic relations between the relevant political communities.^167^ Consequently, any withdrawal of recognition by host states would significantly affect the capacity of sending states to intervene on their nationals’ behalf. The implication of this within the context of sea-level rise is striking. According to the fiduciary model, were submerged SIDS no longer recognised as states, it would not be just the physical fact of their submergence that hampered their effectiveness, but also the social fact of non-recognition itself. This holds because, on fiduciary conceptions of statehood, what makes governance effective is not factual control as such, but the extent to which basic human rights are guaranteed: a broader metric under which the stable governance of inhabitable land is just one contributing factor.^168^ To withdraw recognition from submerged states would therefore constitute a performative act. Rather than merely *declaring* their lack of effectiveness, non-recognition would help *constitute* it as a legal and political reality.^169^

The force of this point is best understood in relation to statelessness. Although the precarity of stateless persons arises partly from their physical displacement,^170^ this is not its only cause. The absence of legal protections for such individuals is both crucial and exacerbated by the unique context of sea-level rise.^171^ International law currently possesses no formal protections for individuals displaced across borders due to climate change.^172^ In particular, according to the ‘dominant view’ of the Refugee Convention,^173^ populations forced into migration due to climate change do not qualify for refugee status independently from other triggering factors.^174^ Even if a displaced individual were to qualify on the basis that they would face persecution in their home state,^175^ non-refoulement has yet to be applied in response to climate change.^176^ Submerging states will likely struggle to protect their nationals abroad even if they continue to be recognised, in part due to losses in infrastructure and resulting reductions of tax revenue.^177^ However, the extent of the lacuna facing individuals displaced by climate change renders the ongoing availability of diplomatic protection effectively essential if the human rights of diasporic populations are to be secured. Since the availability of such protection characteristically requires the ongoing recognition of statehood, the fiduciary model arguably supports state continuity notwithstanding sea-level rise, at least until diasporic populations secure alternative nationalities (again, assuming that this is something that a sufficient number desire).

This point holds equally for international representation, insofar as statehood is necessary for membership of particular international organisations, including the UN.^178^ Although governmental participation within bodies such as the General Assembly or the Security Council cannot secure individual rights directly, the fiduciary model nonetheless takes participation within them to be constitutive of effective statehood because international institutions constitute ‘indirect fiduciaries for humanity’.^179^ Specifically, insofar as they fulfil globally co-ordinative, co-operative, and therefore empowering functions, participation within international organisations allows states to influence transnational issues, such as climate change, which would be impossible for them to address alone.^180^ Conversely, insofar as they are excluded from such organisations, states are disempowered to work on behalf of their populations in a fiduciary capacity.^181^ Since state continuity would uncontroversially result in ongoing membership—and therefore both participation and empowerment—the fiduciary model must once more come down in favour of existential resilience.

## 5. Statehood as Political Community

My final reconstruction emphasises the gestalt quality of states. It casts legal statehood as a status that respects (and protects) the role of states as communities within which private individuals undertake ethically valuable political action.^182^ Such action is valuable because it provides opportunities for authentic self-authorship and the fulfilment of discretely political duties that we owe to our compatriots.^183^ To quote Arendt:

In acting and speaking, men show who they are … This disclosure of ‘who’ in contradistinction to ‘what’ somebody is—his qualities, gifts, talents, and shortcomings, which he may display or hide—is implicit in everything somebody says and does. It can be hidden only in complete silence and perfect passivity …^184^

When politics is understood as an expression of this sort, states matter because they both facilitate and are formed by action of this kind. They are, as I describe below, artefacts, focuses and forums of political action. Conceptions of effectiveness that adopt this view share features of both effectiveness as stability and the fiduciary model. In line with the former, they emphasise the importance of peace insofar as individual political action is impossible amidst endemic violence.^185^ As regards the latter, they share an emphasis upon plausible guarantees of human rights protection as one means through which states can establish effectiveness.^186^ As such, notwithstanding the emphasis it places upon the traditional requirements of factual control,^187^ ‘statehood as political community’ understands effectiveness to demand more than the social fact of territorial governance alone. In what remains, I apply this conception to state continuity and sea-level rise, first by explaining how states facilitate political action and second by demonstrating how submerged SIDS might continue to do so.

### A. Artefacts, Focuses and Forums

Contemporary political communities are very large: the Republic of India comprises well over one billion people, and even the smallest states have populations of well over 10 thousand.^188^ Groups of this size cannot organise themselves on a purely interpersonal basis. Even allowing for internet-based communication, individual humans cannot absorb the vast amount of information that this would require.^189^ Print, digital and social media facilitate mass communication, but politics requires more: we must have ‘something to talk *about* and act *in relation to*’ [emphasis in original].^190^ The conduct of our governments provides this substance, thereby acting as a co-ordinating ‘focus’ for ongoing political action. As I have put the point elsewhere:

Communities with functioning governments and visible institutions are more likely to converge in agreement and disagreement than they would otherwise. This value exists even in circumstances of relative autocracy. Dictators and dominant classes provide visible political targets for protestors, reformers and revolutionaries: consider the internal political opposition to the government of President Zine El Abidine Ben Ali in Tunisia. The mere visibility of such regimes cannot make them just or legitimate, but it would be wrong to dismiss their value completely whilst they operate as effective political focuses.^191^

In addition to focuses, politics requires ‘forums’: physical, institutional or virtual spaces within which individuals can engage politically. Contemporary states provide forums wherever they facilitate popular participation. To take one characteristic example, Part III of the Constitution of the Democratic Republic of Timor-Leste establishes various participatory mechanisms, including a National Parliament subject to popular election.^192^ Even where participation is not afforded in this way, political forums are facilitated whenever states support social environments conducive to politics, whether by providing reasonably effective *de jure* entitlements to free speech and association or by supporting resource distributions conducive to the creation of non-governmental political forums.

Turning to ‘artefacts’, individual political actions are ephemeral: once undertaken, they become a matter of history. Seen in this light, politics is a performance.^193^ By contrast, the institutions that arise in response to established traditions of political action have relative permanence: councils, commissions and parliaments endure insofar as their constitutive practices sustain them.^194^ Moreover, as ‘expression[s] … of the political’,^195^ such institutions ‘stand as intrinsically valuable monuments to whatever individual political activity helped “fix” their existence’.^196^ For example, the fall of apartheid in South Africa in 1994 can only be properly comprehended against a lengthy and diffuse political background of strikes, boycotts and civil disobedience.^197^ Each participant in that action can count the 1990–93 negotiations as a success in which they played an active role: ‘For every Martin Luther King Jr., there are multitudes who contribute in less visible ways, without which power and influence would be impossible.’^198^ Since they are causally downstream from individual political activity, such institutions possess intrinsic value in much the same way that works of art or jokes possess such value, insofar as the latter are artefacts of artistic creation or exhibitions of humour.^199^ They are, in this sense, artefacts of the political traditions they represent: to respect them is to respect the complex histories of participation that imbue them with the forms they currently possess.

Statehood as political community claims that the artefactual, focusing and forum-facilitating nature of governance is endemic to contemporary statehood, in that the vast preponderance of established states provide all three.^200^ Moreover, it understands effectiveness primarily in terms of this provision, the fact of which renders states ‘effective catalysts for political action’.^201^ An important implication of this view is that entities with factual control of inhabitable territory that nonetheless make politics impossible, say, because they are unusually tyrannical or incompetent, cannot be deemed fully ‘effective’.^202^ For this reason, like the fiduciary model, statehood as political community coheres with international practice such as the 1991 EC Guidelines and the collective non-recognition of the Turkish Republic of Northern Cyprus, Southern Rhodesia and the Bantustans. It also fits practice that treats statehood as the primary means for collective self-determination, on the basis that ‘peoples’ should be defined in civic terms.^203^ Such practice includes the Friendly Relations Declaration, which emphasises this role on the part of ‘sovereign and independent States conducting themselves in compliance with the principle of equal rights and self-determination of peoples’,^204^ as well as *Reference re Secession of Quebec*, which held that in ordinary ‘circumstances, peoples are expected to achieve self-determination within the framework of their existing state’.^205^ According to statehood as political community, such practice makes normative sense because our political duties are determined by what we owe to our extant compatriots, who are defined as sets by the historically contingent fact of the states that demarcate them as distinct populations.^206^ This matters, as I argue below, because historical contingency has created various political traditions worthy of ongoing recognition, notwithstanding the threat that many of them face from rising sea-levels.

### B. Political Community and Sea-level Rise

When considering effectiveness and sea-level rise from the perspective of political community, one important question is the extent to which the governance of an autonomous maritime territory could be responsive to the political action and ethos of a diasporic population. If the politics of such populations can maintain strong links with physically removed territorial units, the case for continued effectiveness will be firm, notwithstanding any lack of permanent residence. On the one hand, inhabitable land provides physical forums for political action, meaning that resident populations are arguably better placed than diasporas.^207^ On the other, the existence of overseas territories, such as the Falkland Islands, the Tokelau Islands and French Polynesia, demonstrates that governments, at least, are perfectly capable of exacting considerable influence upon territorial units geographically removed from their primary landmasses. In a similar vein, international law has long recognised the existence of governments in exile:^208^ a point recently raised by states, as well as members of the ILC, to cast doubt on whether the Montevideo criteria of government, population and territory must spatially coincide for state continuity to pertain.^209^ Such argumentative moves sometimes provoke scepticism, usually motivated by fiduciary or stability-related concerns, alleging that overseas rule requires considerable resources that SIDS presently lack and that governments in exile are characterised by relative impotence.^210^

Nonetheless, the governance of autonomous maritime territory could be both constitutive of and responsive to diasporic politics. Taking the constitutive point, maritime governance demonstrably provides a political focus. Many SIDS already identify as ‘large ocean states’,^211^ and have established histories of progressive politics in relation to those oceans.^212^ Taking responsiveness, the very same political initiatives also demonstrate how maritime governance can possess artefactual value. For instance, within international relations, SIDS possess an influence on ocean governance disproportionate to their geographical size and economic power.^213^ Such traditions of international relations provide discrete focuses for political action and can exhibit unique artefactual value,^214^ as evidenced by the recent initiative from Vanuatu requesting an ICJ advisory opinion on climate change, which was developed in response to political action by Pacific Island youth groups.^215^ Such histories of political action make it reasonable to suppose that both artefactual value and political focuses could be generated by SIDS lacking inhabitable land but nonetheless possessing an autonomous maritime territory. This suggests that, according to statehood as political community, effectiveness should be understood capaciously enough to admit governance of this kind.

Space for this capacious reconstruction can be found in international practice. *Western Sahara* held that, notwithstanding the nomadic nature of the peoples within Rio de Oro and Sakiet El Hamra, that territory could not be considered unoccupied.^216^ Two points here are of note. First, when holding ‘State practice of the relevant period indicates that territories inhabited by tribes or peoples having a social and political organization were not regarded as *terrae nullius*’,^217^ the ICJ emphasised that ‘at the time of colonization Western Sahara was inhabited by peoples which, if nomadic, were socially and politically organized in tribes and under chiefs competent to represent them’.^218^ Second, when considering various competing title claims over that territory, the Court noted the

very special characteristics which, at the time of colonization by Spain, largely determined the way of life and social and political organization of the peoples inhabiting [Rio de Oro and Sakiet El Hamra]. In consequence, the legal régime of Western Sahara, including its legal relations with neighbouring territories, cannot properly be appreciated without reference to these special characteristics.^219^

These points matter for two reasons. First, they suggest that, under certain circumstances, nomadic peoples generate territorial title over the lands through which they travel, satisfying the requirements of factual effectiveness notwithstanding their non-settled status. If non-settled status is sufficient to evince effective control in the case of nomadic peoples, it should arguably also be sufficient for diasporic peoples that nonetheless continue to govern maritime territory on a remote basis. This point is all the more convincing, one might think, given the historical precedents of oceanic nomadism set by several Indigenous peoples.^220^

Second, these points from *Western Sahara* demonstrate that when ascertaining effectiveness, the physical geography of the relevant territory must be considered. This can be confirmed by reference to *Legal Status of Eastern Greenland*, which referenced ‘the Arctic and inaccessible character of the uncolonized parts of the country’ when identifying the initial extent of Danish title.^221^*Eastern Greenland* also established that if territorial title is uncontested by another state, the standard for evincing effective control will be lower.^222^ In a similar vein, what holds in relation to deserts should also hold for the ocean, assuming that some other physical or historical basis can be found for maintaining title (in the former case, nomadic routes, sacred and other sites or contiguity with areas of undisputed factual control; in the latter, an erstwhile landmass and continuing governance that exhibits political value).

Turning from artefacts and focuses to forums, it might be supposed that, unlike the former two, the latter necessitates at least *some* inhabitable land.^223^ Nonetheless, it remains conceivable that wholly maritime states, and even states with no territory at all, could provide forums of the relevant kind. The key example here is Tuvalu, which recently announced its aim to create a virtual space within which its culture and politics can endure.^224^ ‘Our digital nation’, according to Prime Minister Natano, ‘will provide an online presence that can replace our physical presence and allow us to continue to function as a state.’^225^ Although it remains to be seen how this proposal will be fleshed out, the ubiquitous nature of political action online, for example through Facebook or Twitter, suggests that, if executed successfully, Tuvalu’s plan could provide a paradigmatic forum for politics.

As noted, this potential extends beyond cementing the political character of purely maritime states and implicates the value that might exist within wholly non-territorial communities. Even purely virtual forums could reinforce the artefactual value of governance undertaken by submerged SIDS, assuming that such governance is appropriately responsive to the online action of their diasporic populations. Moreover, the mere existence of a ‘digital nation’ may provide a focus around which diasporic politics could effectively converge. Given this potential, statehood as political community seemingly supports the continuity of submerged states, whether their maritime territory is retained or not. To quote a recent submission of Antigua and Barbuda to the ILC:

It would be untenable for a people who have already expressed its right to self-determination through statehood, to have that statehood cease in a manner that is imposed upon them. Statehood should only cease if another form of expression of the right to self-determination was explicitly sought by the people who are entitled to exercise their right to self-determination.^226^

‘Uploading’ submerging states to cyberspace may prevent the loss of their political communities and safeguard their self-determination, assuming that statehood continues to be recognised. Statehood as political community shows why such recognition should continue, by illustrating how ‘effective statehood’ might endure in these circumstances.

## 6. Conclusion

Alleging any connection between the existence of legal statehood and some notion of ‘effectiveness’ naturally invites the question, ‘Effective at what?’ The responses most frequently offered within international legal practice sidestep the issue by gesturing in somewhat vague and abstract terms to various properties that established states typically possess. Whether one focuses upon the Badinter Commission’s equation of statehood with ‘a territory and a population subject to an organized political authority’^227^ or the more obviously circular approach of the Permanent Court of Arbitration in *Island of Palmas*,^228^ this quite understandable question goes largely unanswered. In this article, I outlined three possible answers, each based on a different ‘rational reconstruction’ of the relevant law. Effectiveness as stability, motivated by the value of peace and friendly relations, emphasises territorial stability and characterises the principle of effectiveness as partly constitutive of a broad scheme of legal norms geared towards conflict prevention and resolution. The fiduciary model focuses upon the importance of protecting human rights and characterises effectiveness in terms of a state’s ability to provide securing, individual flourishing and civic equality for their populations. Finally, statehood as political community emphasises the role of states as political catalysts and characterises effectiveness in terms of the facilitation of individual political action through the provision of artefactual value, focuses and forums.

Having established these reconstructions, I adopted a broadly teleological approach to the issue of state continuity and sea-level rise, ultimately arguing that, irrespective of which reconstruction one prefers, effectiveness, when understood in relation to its underlying rationale(s), is not only capacious enough to admit submerged SIDS, but also positively *requires* state continuity notwithstanding the total loss of inhabitable territory. By advancing such explicitly ‘non-positivist’ reconstructions of international law within what Lauterpacht and others once called the ‘Grotian tradition’,^229^ my aim has been not only to advance our understanding of legal statehood as such, but also to push its conceptual boundaries for the benefit of states facing significant existential threats. Naturally, this motivation is partly ethical and political, as well as intellectual, but that is how it should be. ‘Large ocean states’ did not cause the global climate crisis. However, unless international lawyers are prepared to undertake the kind of critically normative, reconstructive work I have advanced here, we are dooming them to the inevitability of becoming its most infamous victims.

## Footnotes

1 James Crawford, *The Creation of States in International Law* (OUP 2007) 55–9, 89, 667–9; Alex Green, *Statehood as Political Community: International Law and the Emergence of New States* (CUP 2024) 225–9.

2 Rowan Nicholson, *Statehood and the State-Like in International Law* (OUP 2019) 127–42; Jure Vidmar, *Democratic Statehood in International Law: The Emergence of New States in Post-Cold War Practice* (Hart Publishing 2013) 43–6, 53–4; Crawford, *The Creation of States* (n 1) 93; Green, *Statehood as Political Community* (n 1) 103–11.

3 Crawford, *The Creation of States* (n 1) 329–73; Green, *Statehood as Political Community* (n 1) 78–80.

4 Green, *Statehood as Political Community* (n 1) 128–9; Vidmar (n 2) 53, 238–9.

5 Green, *Statehood as Political Community* (n 1) 82–5; cf Crawford, *The Creation of States* (n 1) 148–55; Nicholson (n 2) 175–80.

6 Green, *Statehood as Political Community* (n 1) 111–15; Vidmar (n 2) 172–4, 247–8.

7 Montevideo Convention on the Rights and Duties of States (adopted 26 December 1933, entered into force 26 December 1934) 165 LNTS 19, Art 1; *Conference on Yugoslavia, Arbitration Commission, Opinion 1* (1991) 92 ILR 162, 164–5; *Deutsche Continental Gas-Gesellschaft v Polish State* (1929) 9 Recueil TAM 336, 344; Crawford, *The Creation of States* (n 1) 45–6, 55–9; Nicholson (n 2) 94–101; Alejandra Camprubí, *Statehood Under Water: Challenges of Sea-Level Rise to the Continuity of Pacific Island States* (Brill 2016) 18–30; cf Green, *Statehood as Political Community* (n 1) 80–5.

8 Crawford, *The Creation of States* (n 1) 59, 89; Thomas Grant, ‘Defining Statehood: The Montevideo Convention and Its Discontents’ (1999) 37(2) Colum J Transnat’l L 403, 409–20.

9
*Island of Palmas Case* (or Miangas) (United States v Netherlands) (Award) (1928) II RIAA 829, 840; Robert Jennings, *The Acquisition of Territory in International Law* (Manchester UP 2017) 33–7.

10 Krystyna Marek, *Identity and Continuity of States in Public International Law* (Librairie E Droz 1954) 1–6.

11 ibid 6.

12 Crawford, *The Creation of States* (n 1) 669–71.

13 This is currently being debated by the International Law Commission. See International Law Commission, ‘Second Issues Paper on Sea-level Rise in Relation to International Law’ (18 April–3 June and 4 July–5 August 2022) UN Doc A/CN.4/752, 21–56; International Law Commission, ‘Report of the Study Group on Sea-Level Rise in Relation to International Law’ (15 July 2022) UN Doc A/CN.4/L.972.

14 Alex Green, ‘*The Creation of States* as a Cardinal Point: James Crawford’s Contribution to International Legal Scholarship’ (2022) 10(1) Aust YBIL 67, 81–3. For endorsements, see Marek (n 10) 7; International Law Commission, ‘Report of the International Law Commission on the Work of Its Seventy-third Session’ (18 April–3 June and 4 July–5 August 2022) UN Doc A/77/10, 332–41; International Law Commission, ‘Second Issues Paper’ (n 13) 48–56; Crawford, *The Creation of States* (n 1) 48, 671; Lassa Oppenheim, *International Law*, vol 1 (8th edn, D McKay 1955) 451.

15 Jürgen Habermas, *Moral Consciousness and Communicative Action* (Christian Lenhardt and Shierry Nicholson tr, Polity Press 1992) 29–32; Alex Green, ‘A Political Theory of State Equality’ (2023) 14(2) TLT 178, 188–91.

16 Ronald Dworkin, *Law’s Empire* (Hart Publishing 1986) 50–3.

17 ibid 65–70, 225–75.

18 John Tasioulas, ‘In Defence of Relative Normativity: Communitarian Values and the Nicaragua Case’ (1996) 16(1) OJLS 85, 111–15.

19 Bașak Çali, ‘On Interpretivism and International Law’ (2009) 20(3) EJIL 805, 807–9.

20 Habermas (n 15) 30; Green, ‘A Political Theory’ (n 15) 190.

21 These are considerations that actually count for, or against, particular kinds of behaviour. See Derek Parfit, *On What Matters*, vol 1 (OUP 2011) 31.

22 Dworkin (n 16) 66–72.

23 Hersch Lauterpacht, ‘The Grotian Tradition in International Law’ (1946) 23 BYBIL 1.

24 Green, *Statehood as Political Community* (n 1) 3–12.

25 Evan Criddle and Evan Fox-Decent, *Fiduciaries of Humanity: How International Law Constitutes Authority* (OUP 2016) 45–76. See also Mortimer Sellers, *Republican Principles in International Law: The Fundamental Requirements of a Just World Order* (Palgrave Macmillan 2006) 61–2, 99–102; Fernando Tesón, *A Philosophy of International Law* (Westview Press 1998) 57–66; Patrick Capps, *Human Dignity and the Foundations of International Law* (Hart Publishing 2009) 194–5.

26 Green, *Statehood as Political Community* (n 1) Part I (generally), 225–9.

27 ibid Part II (generally).

28 (1929) 9 Recueil TAM 336, 344.

29 (1991) 92 ILR 162, 164–5.

30 League of Nations Official Journal, Special Supplement 4 (1920) 8–9.

31 Crawford, *The Creation of States* (n 1) 55–6; Green, *Statehood as Political Community* (n 1) 80–1.

32 Crawford, *The Creation of States* (n 1) 59.

33 Nicholson (n 2) 95; Jure Vidmar and Lea Raible, ‘State Creation and the Concept of Statehood in International Law’ in Jure Vidmar, Sarah McGibbon and Lea Raible (eds), *Research Handbook on Secession* (Edward Elgar 2022) 25–6.

34 Crawford, *The Creation of States* (n 1) 61–2.

35 ibid 62–89; Green, *Statehood as Political Community* (n 1) 85–98.

36 Institut de Droit International, ‘Resolutions Concerning the Recognition of New States and New Governments’ (1936) 30(4) AJIL 185.

37 United Nations Yearbook of the International Law Commission (1949) 287.

38 Dworkin (n 16) 228–32.

39 Crawford, *The Creation of States* (n 1) 60, 671. This view is not universally held: Antigua & Barbuda, ‘Response to the International Law Commission, Second Issues Paper on Sea Level Rise and the Effect on Statehood and Protection of Persons’ (30 June 2023) UN Doc A/CN.4/752, paras 5–11; Chiara Giorgetti, *A Principled Approach to State Failure* (Martinus Nijhoff 2010) 65–6.

40 Crawford, *The Creation of States* (n 1) 89.

41 Marek (n 10) 15–24.

42 See generally Emizet Kisangani, *Civil Wars in the Democratic Republic of Congo 1960–2010* (Lynne Rienner 2012).

43 Walter Roberts, *Tito, Mihailović, and The Allies, 1941–1945* (Duke UP 1987) 312–13.

44 Crawford, *The Creation of States* (n 1) 692–5.

45 ibid 715–16.

46 ibid 688–90.

47 Green, ‘*The Creation of States*’ (n 14) 82–3.

48 International Law Association, ‘Sydney Conference (2018), International Law and Sea Level Rise: Report’ (2018) 25.

49 International Law Commission, ‘Second Issues Paper’ (n 13) 184–91.

50 International Law Commission, ‘PSIDS Statement: Sixth Committee- Agenda Item 82: Report of the International Law Commission on the Work of its Seventy-Second Session’ (28 October 2021) UN Doc A/C.6/76/SR.19, para.71.

51 Pacific Islands Forum, ‘2023 Declaration on the Continuity of Statehood and the Protection of Persons in the Face of Climate Change-Related Sea-Level Rise’ (9 November 2023) paras 12–13.

52 Green, ‘*The Creation of States*’ (n 14).

53 Crawford, *The Creation of States* (n 1) 48. See also Carolin König, *Small Island States and International Law: The Challenge of Rising Seas* (Routledge 2022) 33–5.

54 Oppenheim, *International Law* (n 14).

55 UN SCOR, 383rd mtg (2 December 1948) UN Doc S/PV.383, 11.

56 (1991) 92 ILR 162, 164–5.

57 Nicholson (n 2) 111.

58 Malcolm Shaw, ‘Territory in International Law’ (2009) 13 Netherlands Journal of International Law 61, 61–3.

59 ‘Sea-level Rise in Relation to International Law: Submission of the United Kingdom of Great Britain and Northern Ireland’ (30 June 2023) para 2 <https://legal.un.org/ilc/sessions/75/pdfs/english/slr_uk.pdf> accessed 23 October 2023; ‘Sea-level Rise in Relation to International law: German Submission to the International Law Commission in Preparation of Its 75th Session (2023)—Answer to Questions Posed in the Commission’s Report of the 73rd Session (2022), Chapter III’ (30 June 2023) 3 <https://legal.un.org/ilc/sessions/75/pdfs/english/slr_germany.pdf> accessed 23 October 2023.

60 International Relations and Defence Committee, *UNCLOS: The Law of the Sea in the 21st Century* (HL 2021–22, 159-2) 34 [117]–[126].

61 Steven Ratner, *The Thin Justice of International Law: A Moral Reckoning of the Law of Nations* (OUP 2015) 184–90. See also Ratner’s assertion that peace is a ‘basic value’ of international law (65).

62 Capps (n 25) 258–64.

63 Lassa Oppenheim, ‘The Science of International Law: Its Task and Method’ (1908) 2(2) AJIL 313, 314; Prosper Weil, ‘Towards Relative Normativity in International Law?’ (1983) 77(3) AJIL 413; Ingrid Wuerth, ‘International Law in the Post-Human Rights Era’ (2017) 96 Texas LR 279.

64 Green, ‘A Political Theory’ (n 15) 185–7.

65 Ratner, *The Thin Justice of International Law* (n 61) 187. See also Capps (n 25) 264–5; Jeremy Waldron, ‘Two Concepts of Self-Determination’ in Samantha Besson and John Tasioulas (eds), *The Philosophy of International Law* (OUP 2010) 407; Allen Buchanan, *Justice, Legitimacy, and Self-Determination: Moral Foundations for International Law* (OUP 2004) 265.

66 Ratner, *The Thin Justice of International Law* (n 61) 188.

67 ibid 197–201.

68 Martti Koskenniemi, *From Apology to Utopia: The Structure of International Legal Argument* (CUP 2005) 17–23.

69 James Crawford, *Chance, Order, Change: The Course of International Law* (Hague Academy of International Law 2014) 31–52.

70 Jack Goldsmith and Eric Posner, *The Limits of International Law* (OUP 2005) 11.

71 Ratner, *The Thin Justice of International Law* (n 61) 198.

72 cf Green, *Statehood as Political Community* (n 1) 161–7.

73 Capps (n 25) 260–1, 264–5.

74 Green, *Statehood as Political Community* (n 1) 122–5; Nicholson (n 1) 166–7.

75 Ratner, *The Thin Justice of International Law* (n 61) 187.

76 Green, *Statehood as Political Community* (n 1) 122–6.

77 Vidmar (n 2) 63.

78 See eg Freddy Mnyongani, ‘Between a Rock and a Hard Place: The Right to Self-determination versus *uti possidetis* in Africa’ (2008) 41(3) Comparative and International Law Journal of Southern Africa 463; Jan Klabbers and René Lefeber, ‘Africa: Lost Between Self-determination and *uti possidetis*’ in Catherine Brölmann, René Lefeber and Marjoleine Zieck (eds), *Peoples and Minorities in International Law* (Brill 1993); Steven Ratner, ‘Drawing a Better Line: *Uti Possidetis* and the Borders of New States’ (1996) 90(4) AJIL 590.

79 Ratner, *The Thin Justice of International Law* (n 61) 173.

80 ibid 176.

81 Green, *Statehood as Political Community* (n 1) 144.

82 Charter of the United Nations (adopted 26 June 1945, entered into force 24 October 1945) 1 UNTS XVI, Art 2(4).

83 Such meta-principles do not entail that more concrete laws never ‘conflict’: factual effectiveness and the presumption of state continuity often do. This matters, to quote Joseph Raz, because ‘we should adopt a doctrine of individuation which keeps laws to a manageable size, avoids repetition, minimizes the need to refer to a great variety of … sources’: Joseph Raz, ‘Legal Principles and the Limits of Law’ (1972) 81(5) Yale LJ 823, 832. However, it *does* imply that conflicts need not be settled by one principle ‘trumping’ the other. To quote again: ‘In determining what is to be done in every case … the importance of the principles has to be considered in relation to the amount of good or harm done to the ends they seek to promote’ (ibid). Under effectiveness as stability, the goal is peace and friendly relations. As argued below, thanks to the meta-principle, more concrete legal principles can be interpreted holistically, rather than being forced to trump one another *ad hoc*.

84
*Case concerning the Temple of Preah Vihear (Cambodia v Thailand) (Merits)* [1962] ICJ Rep 6, 34.

85 United Nations Convention on the Law of the Sea (signed 10 December 1982, entered into force 16 November 1994) 1843 UNTS 397. See also Massimo Lando, ‘Stability of Maritime Boundaries and the Challenge of Geographical Change: A Reply to Snjólaug Árnadóttir’ (2022) 35 LJIL 379, 396.

86 This is echoed in statements by Member States: International Law Commission, ‘Sea-level Rise in Relation to International Law: Additional Paper to the First Issues Paper (2020), by Bogdan Aurescu and Nilüfer Oral, Co-Chairs of the Study Group on Sea-level Rise in Relation to International Law’ (13 February 2023) UN Doc A/CN.4/761, paras 20–76.

87 Roland Rich, ‘Recognition of States: The Collapse of Yugoslavia and the Soviet Union’ (1993) 4 EJIL 56; Victor Kattan, ‘Book Review: Jure Vidmar, Democratic Statehood in International Law: The Emergence of New States in Post-Cold War Practice’ (2015) 78(2) MLR 394, 395–7.

88 Vaughan Lowe, *International Law* (OUP 2007) 163; cf Alex Green, ‘Successful Secession and the Value of International Recognition’ in Vidmar, McGibbon and Raible (n 33) 81–3.

89 Green, *Statehood as Political Community* (n 1) 103–11; Nicholson (n 2) 127–42; Vidmar (n 2) 239–53.

90 Crawford, *The Creation of States* (n 1) 59.

91 Nicholson (n 2) 127–42.

92 Ronald Dworkin, *Justice in Robes* (Harvard UP 2006) 105–16.

93 Sometimes ‘Da’ish’ or ‘Islamic State’. Other grounds for rejecting the statehood of these entities include lack of independence in the case of Donetsk or Luhansk (Green, *Statehood as Political Community* (n 1) 92–3) or the impermanence of Daesh. Permanence—that is, maintaining effectiveness over time—has evidential value but no real support as an independent criterion for statehood: Crawford, *The Creation of States* (n 1) 90–1.

94 UNCLOS, Arts 121(1) and 121(3); Robin Churchill, Vaughan Lowe and Amy Sander, *The Law of the Sea* (4th edn, Manchester UP 2022) 90.

95
*North Sea Continental Shelf (Federal Republic of Germany v Denmark; Federal Republic of Germany v Netherlands* (Merits) [1969] ICJ Rep 3, para 96; Bing Bing Ja, ‘The Principle of the Domination of the Land over the Sea: A Historical Perspective on the Adaptability of the Law of the Sea to New Challenges’ (2014) 57 German Yearbook of International Law 1; Kate Purcell, *Geographical Change and the Law of the Sea* (OUP 2019) 44–8.

96 Local priority, captured by the maxim *lex specialis derogat legi generali*, concerns ‘what we might call “departments” of law’ (Dworkin (n 16) 250), which can be separated out to promote predictability (ibid 252). Observing that local priority complies with states’ expectations is International Law Commission, ‘Fragmentation of International Law: Difficulties Arising from the Diversification and Expansion of International Law: Report of the Study Group of the International Law Commission’ (18 July 2006) UN Doc A/CN.4/L.682, paras 9, 12, 14(5)–(16).

97 International Law Commission, ‘Report of the International Law Commission’ (n 14) 196.

98 Rosemary Rayfuse, ‘Sea Level Rise and Maritime Zones’ in Michael Gerrard and Gregory Wannier (eds), *Threatened Island Nations: Legal Implications of Rising Seas and a Changing Climate* (CUP 2013) 183–5; Camprubí (n 7) 100–3.

99 Churchill, Lowe and Sander (n 94) 49–51.

100 ibid 50, 110–19.

101 I am grateful to Mathias Forteau for suggesting this. See also Alina Miron, ‘Les conséquences de l’élévation du niveau des mers sur le tracé des lignes de base’ in V Boré Eveno (ed), *Elévation du niveau de la mer et droit international. De l’adaptation à l’action* (Pedone 2022) 106–8.

102
*Fisheries Case (United Kingdom v Norway)* (Merits) [1951] ICJ Rep 116, 128; Churchill, Lowe and Sander (n 92) 54–60.

103 Clive Schofield, ‘Rising Waters, Shrinking States: The Potential Impacts of Sea Level Rise on Claims to Maritime Jurisdiction’ (2010) 53 German Yearbook of International Law 189, 224–6; Davor Vida, David Freeston and Jane McAdam, *Report of the International Law Association Committee on International Law and Sea Level Rise* (Brill 2019) 17–20.

104 Churchill, Lowe and Sander (n 94) 50, 141–55.

105 ibid 100–8.

106 Pacific Islands Forum, ‘Declaration on Preserving Maritime Zones in the Face of Climate Change-related Sea-Level Rise’ (6 August 2021).

107 Office of Ocean and Polar Affairs (USA), ‘U.S. Policy on Sea-level Rise and Maritime Zones’ <www.state.gov/marine-environment/#sea-level-rise> accessed 23 October 2023.

108 Abhimanyu George Jain, ‘The 21st Century Atlantis: The International Law of Statehood and Climate Change-Induced Loss of Territory’ (2014) 50 Stan J Int’l L 1, 27. On average, SIDS exclusive economic zones (EEZs) are 2133 times larger than their landmasses, while other coastal states on average have EEZs only six times larger: Iris Monnereau and others, ‘Fisheries Sector Vulnerabilities to Climate Change in Small Island Developing States’ in Stefano Moncada and others (eds), *Small Island Developing States: Vulnerability and Resilience Under Climate Change* (Springer 2021) 235.

109 See generally JE Carroz and AG Roche, ‘The International Policing of High Sea Fisheries’ (1968) 6 Canadian Yearbook of International Law 61; Frank Müller-Rath and Uwe Althaus, ‘Policing the High Seas—A Mission Impossible?’ (2018) 1(3/4) Humanitäres Völkerrecht 209; Michael Byers, ‘Policing the High Seas: The Proliferation Security Initiative’ (2004) 98(3) AJIL 526.

110 Ori Sharon, ‘To Be or Not to Be: State Extinction through Climate Change’ (2021) 51(4) Environmental Law 1041, 1065.

111 ibid 1063–4.

112 ibid (original emphasis).

113 Camprubí (n 7) 113–14, 221; Jain (n 108) 49–51.

114 Sharon (n 110) 1063.

115 Jennings (n 9) 14–20.

116 ibid 56–60.

117
*Island of Palmas Case* (n 9) 840.

118
*Frontier Dispute (Burkina Faso v Mali)* (Merits) 1986 ICJ Rep 554, 586–7: ‘Where the [reality of factual control] does not correspond to law, where the territory which is the subject of the dispute is effectively administered by a State other than the one possessing the legal title, preference should be given to the holder of the title.’ Contrary examples, such as the secession of the People’s Republic of Bangladesh or the State of Kosovo, are better explained in other terms. Bangladesh was either entitled to remedial secession (Green, *Statehood as Political Community* (n 1) 133–6) or constituted a self-determination unit, meriting statehood on that basis (Crawford, *The Creation of States* (n 1) 140–3). Kosovo emerged following the dissolution of SFRY, and what recognition it has received must be understood in that context. Neither case involved the unilateral assertion of title by one established state against another.

119 Crawford, *The Creation of States* (n 1) 417.

120 Jain (n 108) 26–7.

121 Churchill, Lowe and Sander (n 94) 105.

122 Lon Fuller, *The Morality of Law* (Yales UP 1969) 79–81. Note, for example, the two senses of stability used by the first president of UNCLOS III: ‘a convention or conventions ensuring a generally acceptable, stable and durable law of the sea would be not only a monument to the patience, perseverance, diplomatic skill and spirit of fraternal cooperation of the participants and the States they represented, but would also honour the highest ideals of the Charter’ (‘Official Records of the Third United Nations Conference on the Law of the Sea, vol I (Summary Records of Plenary Meetings of the First and Second Sessions, and of Meetings of the General Committee, Second Session)’ UN Doc A/CONF./BUR/SR.1, 4). ‘General acceptability’ implies a *modus vivendi*, which is conducive to peace in a different manner from ‘stable and durable law’.

123 Churchill, Lowe and Sander (n 94) 102–3.

124 Address by Knut Vollebaek, OSCE High Commissioner on National Minorities, to the Expert Consultation on ‘Issues related to minorities and the denial or deprivation of citizenship’, convened by the UN Independent Expert on Minority Issues, Gay McDougall, Geneva, 6 December 2007. See also and generally UN High Commissioner for Refugees, ‘Statelessness and Citizenship’ in *The State of the World’s Refugees—A Humanitarian Agenda* (OUP 1997).

125 William Conklin, *Statelessness: The Enigma of the International Community* (Hart Publishing 2014) 113–17, 126–34.

126 This is always context-dependent: Ted Robert Gurr, *Why Men Rebel* (Princeton UP 1970); Mohammed Hafez, *Why Muslims Rebel: Repression and Resistance in the Islamic World* (Lynne Rienner 2003); Douglas McAdam, *Political Process and the Development of Black Insurgency: 1930–1970* (University of Chicago Press 1982); Raquel Rolnik, ‘Territorial Exclusion and Violence: The Case of the State of São Paulo, Brazil’ (2001) 32 Geoforum 471; Markus Holdo and Bo Bengtsson, ‘Marginalization and Riots: A Rationalistic Explanation of Urban Unrest’ (2020) 37(2) Housing, Theory and Society 162.

127 Crawford, *The Creation of States* (n 1) 52–3.

128 Jane McAdam, *Climate Change, Forced Migration, and International Law* (OUP 2012) 136; Maxine Burkett, ‘The Nation Ex-Situ’ in Gerrard and Wannier (n 98) 107–20.

129 Pacific Islands Forum, ‘Boe Declaration Action Plan’ (2018) 10 <www.forumsec.org/wp-content/uploads/2019/10/BOE-document-Action-Plan.pdf> accessed 23 October 2023. See also Permanent Representative of Malta to the United Nations, ‘Letter Dated 2 February 2023, Addressed to the Secretary-General’ (2 February 2023) UN Doc S/2023/79; Polynesian Leaders Group, ‘Polynesia Against Climate Threats: Taputapuātea Declaration on Climate Change’ (16 July 2015) 3.

130 Office of the High Representative for the Least Developed Countries, Landlocked Developing Countries and Small Island Developing States, ‘About Small Island Developing States’ <www.un.org/ohrlls/content/about-small-island-developing-states> accessed 23 October 2023; UN Refugee Agency, ‘Statelessness Around the World’ <www.unhcr.org/ibelong/statelessness-around-the-world/> accessed 23 October 2023.

131 Initial displacement will likely be internal: Miriam Cullen, ‘Disaster, Displacement and International Law: Legal Protections in the Context of a Changing Climate’ (2020) 8(4) Politics and Governance 270, 274.

132 Melissa Stewart, ‘Cascading Consequences of Sinking States’ (2023) 59(2) Stan J Int’l L 131, 170.

133 This is acknowledged within Art 2(1) of the Australia–Tuvalu Falepili Union Treaty (signed 9 November 2023, not yet in force), which references ‘stability, security, prosperity and resilience’. Art 2(2)(b) of that treaty reads: ‘the statehood and sovereignty of Tuvalu will continue, and the rights and duties inherent thereto will be maintained, notwithstanding the impact of climate change-related sea-level rise’.

134 Criddle and Fox-Decent (n 25) 45–7, 50–2.

135 Green, *Statehood as Political Community* (n 1) 235.

136 ibid 234.

137 Stefan Talmon, *Recognition of Governments in International Law—with Particular Reference to Governments in Exile* (OUP 1998) 3–14.

138 Crawford, *The Creation of States* (n 1) 671.

139 Barring difficult cases (Talmon (n 137) 89–111), it is legally uncontroversial that governments speak for their states by ‘acting in their place’ (Hanna Pitkin, *The Concept of Representation* (California UP 1967) 82). The point is not that governments are *actually* representative of those they govern (which might justify this), but that they act *as though they were* (ibid).

140 Criddle and Fox-Decent (n 25) 52.

141 Jeremey Waldron, ‘Are Sovereigns Entitled to the Benefit of the International Rule of Law?’ (2011) 22(2) EJIL 315, 328–37. For the importance of resisting such elision, see Green, *Statehood as Political Community* (n 1) 170–8.

142 Tesón (n 25); Sellers (n 25); Samantha Besson, ‘International Courts and the Jurisprudence of Statehood’ (2019) 10(1) TLT 30.

143 Andrew Altman and Christopher Heath Wellman, *A Liberal Theory of International Justice* (OUP 2009) 12–16; Buchanan (n 63) 266–75; Margaret Moore, *A Political Theory of Territory* (OUP 2015) 50, 62; Cara Nine, *Global Justice and Territory* (OUP 2012) 67; Anna Stilz, *Territorial Sovereignty: A Philosophical Exploration* (OUP 2019) 20–3.

144 European Community: Declaration on Yugoslavia and on the Guidelines on the Recognition of New States (1992) 31 ILM 1485, 1487.

145 Charter of Paris for a New Europe (1991) 30 ILM 193, 194. When applying these standards to the states emerging after the dissolution of SFRY, the Badinter Commission focused mainly upon the text of various constitutional provisions: *Conference on Yugoslavia, Arbitration Commission, Opinion 5* (1992) 92 ILR 188; *Opinion 6* (1992) 92 ILR 182; *Opinion 7* (1992) 31(6) ILM 1512.

146 James Fawcett, ‘Security Council Resolutions on Rhodesia’ (1965) 41 BYBIL 103, 112–13; Green, *Statehood as Political Community* (n 1) 83–5.

147 Crawford, *The Creation of States* (n 1) 128–31, 338–47; Criddle and Fox-Decent (n 25) 68–9; Fawcett (n 146); Green, *Statehood as Political Community* (n 1) 116–22; WM Reisman, ‘Private Armies in a Global War System: Prologue to Decision’ (1973) 14(1) Va J Int’l L 1, 4.

148 Crawford, *The Creation of States* (n 1) 148–55.

149 Capps (n 25) 264–5.

150 Criddle and Fox-Decent (n 25) 51.

151 On ideal types, see Max Weber, *The Methodology of the Social Sciences* (EA Shils and HA Finch, ed and tr, Free Press 1949) 90.

152 Capps (n 25) 264.

153 ibid 265. Similar language appears in Criddle and Fox Decent (n 25) 76: ‘As juridical institutions constituted by international law, states have no authority to use their sovereign powers for any purpose other than to advance the well-being of their people … The fiduciary conception of the state thus provides a united standard of authorization that permits critical scrutiny and regulation …’

154 Criddle and Fox Decent (n 25) 52.

155 Capps (n 25) 194: ‘the moral value of the sovereign state to its subjects is the sole reason why states can be said to have rights as a sovereign state’.

156 ibid.

157 Mortimer Sellers, ‘Republican Principles in International Law’ (1996) 11(3) Connecticut Journal of International Law 403, 432.

158 As argued by the International Law Association in their 2022 Lisbon Conference Final Report: ‘the more fragmented the location of the population and government, the more exposed they are to the sovereignty of one or more other States, the more the State’s ability to perform the core functions of a State or to exercise sovereign power over its population, including the protection of human rights, is compromised’ (23).

159 On the former, see Elihu Lauterpacht and Daniel Bethlehem, ‘The Scope and Content of the Principle of Non-refoulement: Opinion’ in Erika Feller, Volker Türk and Frances Nicholson (eds), *Refugee Protection in International Law* (CUP 2009) 121–2. On the latter, see *Banković v Belgium*, App no 52207/99 (Decision, 12 December 2001) para 59; Michal Gondek, ‘Extraterritorial Application of the European Convention on Human Rights: Territorial Focus in the Age of Globalization?’ (2005) 52 NILR 349, 360; Samantha Besson, ‘The Extraterritoriality of the European Convention on Human Rights: Why Human Rights Depend on Jurisdiction and What Jurisdiction Amounts to’ (2012) 25(4) LJIL 857, 876; Lea Raible, *Human Rights Unbound: A Theory of Extraterritoriality* (OUP 2020) 176–80.

160 eg International Law Commission, ‘Draft Articles on Diplomatic Protection with Commentaries’ (9 August 2006) UN Doc A/61/10, Art 1.

161 Talmon (n 137) 202.

162
*Mavrommatis Palestine Concessions* (1924) PCIJ Series A, No 2, 12.

163 eg *Ahmadou Sadio Diallo (Republic of Guinea v Democratic Republic of the Congo)* (Preliminary Objections) [2007] ICJ Rep 582, para 42.

164 Vienna Convention on Diplomatic Relations of States (signed 18 April 1961, entered into force 24 April 1964) 500 UNTS 95, Art 3(1)(b).

165 See n 162.

166 Guy Leigh, ‘Nationality and Diplomatic Protection’ (1971) 20(3) ICLQ 453.

167 Talmon (n 137) 203.

168 Criddle and Fox-Decent (n 25) 67.

169 As such, the fiduciary model typically rejects ‘constitutive’ and ‘declaratory’ theories of recognition in favour of ‘deliberative’ understandings. See ibid 71–5. For traditional approaches, see Crawford, *The Creation of States* (n 1) 3–36.

170 International Law Association (n 158).

171 Conklin (n 125) 113–34.

172 Susan Martin, ‘Climate Change, Migration, and Governance’ (2010) 16(3) Global Governance 397, 403.

173 Convention Relating to the Status of Refugees (signed 28 July 1951, entered into force 22 April 1954) 189 UNTS 137, Art 1(a)(2).

174 Matthew Scott, *Climate Change, Disasters, and the Refugee Convention* (CUP 2020) 1; UN High Commissioner for Refugees, ‘Handbook on Procedures and Criteria for Determining Refugee Status under the 1951 Convention and the 1967 Protocol Relating to the Status of Refugees’ (2019) UN Doc HCR/1P/4/ENG/REV. 4, 13.

175 UN High Commissioner for Refugees, ‘Legal Considerations Regarding Claims for International Protection Made in the Context of the Adverse Effects of Climate Change and Disasters’ (1 October 2020) paras 6, 64–106.

176 Jane McAdam, ‘Protecting People Displaced by the Impacts of Climate Change: The UN Human Rights Committee and the Principle of “Non-Refoulement”’ (2020) 114(4) AJIL 708, 712–13. This was considered by the UN Human Rights Committee in: ‘Views Adopted by the Committee Under Article 5(4) of the Optional Protocol, Concerning Communication No. 2728/2016’ (23 September 2023) UN Doc CCPR/C/127/D/2728/2016. Although the Committee held that non-refoulement might be triggered by climate-related threats, they did not consider the facts in that complaint sufficient to do so (ibid para 9.11). They emphasised that the Republic of Kiribati would likely not become uninhabitable for at least the next 10 years: ibid para 9.12. The fiduciary model *may* support the extension of non-refoulement to climate-displaced diasporas, since it takes an ‘absence of protection’ to trigger that principle: Criddle and Fox-Decent (n 25) 271–2. However, most contemporary accounts emphasise ‘persecution’, which disqualifies many individuals who might otherwise be considered ‘climate refugees’: ibid 272–5.

177 Republic of Maldives, *Maldives Submission Under Human Rights Council Resolution 7/23* (25 September 2008) 19; Government of Marshall Islands, *Adaptation Communication—Republic of Marshall Islands (RMI)* (December 2020) 10; Eleanor Ainge Roy, ‘“One Day We’ll Disappear”: Tuvalu’s Sinking Islands’ *The Guardian* (16 May 2019) <www.theguardian.com/global-development/2019/may/16/one-day-disappear-tuvalu-sinking-islands-rising-seas-climate-change> accessed 23 October 2023.

178 UN Charter, Art 4 (1); *Admission of a State to the United Nations (Charter, Art. 4) (Advisory Opinion)* [1948] ICJ Rep 57, 62; Rossalyn Higgins, *The Development of International Law Through the Political Organs of the United Nations* (OUP 1963) 11–57.

179 Criddle and Fox-Decent (n 25) 290.

180 ibid 290–2.

181 One recent example is UNGA, ‘Protection of Civilians and Upholding Legal and Humanitarian Obligations’ (30 October 2023) UN Doc A/RES/ES-10/21. This resolution concerned the ongoing conflict in and around the Gaza Strip. The State of Palestine was implicated without being empowered to vote. See further and generally Shadi Sakran, *The Legal Consequences of Limited Statehood: Palestine in Multilateral Frameworks* (Routledge 2020).

182 Green, *Statehood as Political Community* (n 1) 1; Green, ‘A Political Theory’ (n 15) 192–8.

183 Green, *Statehood as Political Community* (n 1) 19–69; Alex Green, ‘Three Models of Political Membership: Delineating “The People in Question”’ (2021) 41(2) OJLS 565, 577–85.

184 Hannah Arendt, *The Human Condition* (Chicago UP 1958) 179.

185 Green, *Statehood as Political Community* (n 1) 52–6.

186 ibid 84.

187 ibid 75–8, 82–3.

188 The Republic of Nauru and Tuvalu both have populations of around 11,200: Nauru Bureau of Statistics website <http://nauru.prism.spc.int/> accessed 23 October 2023; ‘Country Facts’, Permanent Mission of Tuvalu to the United Nations website <www.un.int/tuvalu/tuvalu/country-facts> accessed 23 October 2023.

189 Anthony Lincoln, ‘FYI: TMI: Toward a Holistic Social Theory of Information Overload’ (2011) 16(3) First Monday <http://firstmonday.org/ojs/index.php/fm/article/view/3051/2835> accessed 23 October 2023.

190 Green, *Statehood as Political Community* (n 1) 64.

191 Green, ‘A Political Theory’ (n 15) 197.

192 Democratic Republic of Timor-Leste, *Constituição da República Democrática de Timor-Leste* (20 March 2022) Part III, Titles I and III.

193 John Parkinson, *Democracy and Public Space: The Physical Sites of Democratic Performance* (OUP 2012) 23.

194 Green, *Statehood as Political Community* (n 1) 61–2.

195 Martin Loughlin, *The Foundations of Public Law* (OUP 2010) 194, citing Georg Jellinek, *Allgemeine Staatslehre* (3rd edn, Springer 1922) 180.

196 Green, *Statehood as Political Community* (n 1) 61.

197 South Africa: Overcoming Apartheid Building Democracy, ‘The Defiance Campaign’ <http://overcomingapartheid.msu.edu/multimedia.php?id=65-259-9> accessed 23 October 2023.

198 Green, *Statehood as Political Community* (n 1) 43. See also David Hume, *Essays, Moral, Political, and Literary* (Eugene Miller ed, Liberty Fund 1987) I.IV.1; Vladimir Ilich Lenin, *Lenin, Selected Works*, vol 7 (Progress Publishers 1961) 295; Loughlin (n 195) 81–3.

199 Green, *Statehood as Political Community* (n 1) 62–3.

200 ibid 61–7, 70–100.

201 ibid 84.

202 This applies only to extreme cases like Daesh or the People’s Democratic Republic of North Korea (ibid). See also Philip Pettit, *The State* (Princeton UP 2023) 61–2.

203 Green, ‘Three Models of Political Membership’ (n 183). See also Waldron (n 63) 406–13.

204 UNGA Res 2625 (XXVI) (24 October 1970) UN Doc A/RES/2625(XXV), Principle 5.

205
*Reference re Secession of Quebec* [1998] 2 SCR 217 (Canada), para 154. This language was adopted almost verbatim in *Reference by the Lord Advocate of devolution issues under paragraph 34 of schedule 6 of the Scotland Act 1998* [2022] UKSC 31 (UK), paras 88–9.

206 Green, ‘Three Models of Political Membership’ (n 183) 583–5.

207 Green, *Statehood as Political Community* (n 1) 78.

208 Talmon (n 137) 115–16.

209 International Law Commission, ‘Second Issues Paper’ (n 13) paras 138–54; International Law Commission, ‘Report of the Study Group on Sea-Level Rise’ (n 13) para 213(b); Antigua & Barbuda (n 39) para 40.

210 Stewart (n 132) 135, 151, 169–70.

211 Nicholas Chan, ‘“Large Ocean States”: Sovereignty, Small Islands, and Marine Protected Areas in Global Oceans Governance’ (2018) 24 Global Governance 537, 540–5.

212 eg Rebecca Gruby and Lisa Campbell, ‘Scalar Politics and the Region: Strategies for Transcending Pacific Island Smallness on a Global Environmental Governance Stage’ (2013) 45(9) Environment and Planning A 2027; Justin Alger, *Conserving the Oceans: The Politics of Large Marine Protected Areas* (OUP 2021) 134–58; Michelle Scobie, *Global Environmental Governance and Small States: Architectures and Agency in the Caribbean* (Edward Elgar 2019) 1–13, 90–117; Victoria Syddalla, Karen Fisher and Simon Thrush, ‘Collaboration a Solution for Small Island Developing States to Address Food Security and Economic Development in the Face of Climate Change’ (2022) 221 Ocean and Coastal Management 1.

213 James Crawford, ‘Islands as Sovereign Nations’ (1989) 38(2) ICLQ 277; Tom Long, *A Small State’s Guide to Influence in World Politics* (OUP 2022) 144–50.

214 Green (n 88) 81–90.

215 Pacific Island Students Fighting Climate Change, ‘We Are the Alliance for a Climate Justice Advisory Opinion’ <www.pisfcc.org/alliance> accessed 23 October 2023.

216
*Western Sahara* (Advisory Opinion) [1975] ICJ Rep 12, para 81.

217 ibid 80.

218 ibid 81.

219 ibid 87.

220 One illustrative example is the Sama-Bajau peoples within Southeast Asia: Rodney Jubilado, ‘On Cultural Fluidity: The Sama-Bajau of the Sulu-Sulawesi Seas’ (2010) 32(1) Kunapipi 89; Rodney Jubilado, Hanafi Hussin and Maria Manueli, ‘The Sama-Bajaus of the Sulu-Sulawesi Seas: Perspectives from Linguistics and Culture’ (2010) 15 Jati 83.

221
*Legal Status of Eastern Greenland, Denmark v Norway*, Judgment, PCIJ Series A/B No 53, 50–1.

222 ibid 46.

223 Green, *Statehood as Political Community* (n 1) 78.

224 Natalie Jonas, ‘As Tuvalu Succumbs to Rising Sea Levels, the Island Nation Wants to Be the First Virtue Country’ *Salon* (3 December 2022): <www.salon.com/2022/12/03/as-tuvalu-succumbs-to-rising-sea-levels-the-island-nation-wants-to-be-the-first-virtual-country/> accessed 23 October 2023.

225 ibid.

226 Antigua & Barbuda (n 39) para 27.

227 See n 56.

228 Above (n 9) 893.

229 Lauterpacht (n 23).

